# Energy Level Tuning in Conjugated Donor Polymers by Chalcogen Exchange for Low Dark Current Organic Photodetectors

**DOI:** 10.1021/acsmaterialslett.4c01899

**Published:** 2024-10-08

**Authors:** Martina Rimmele, Zhuoran Qiao, Filip Aniés, Adam V. Marsh, Aren Yazmaciyan, George Harrison, Shadi Fatayer, Nicola Gasparini, Martin Heeney

**Affiliations:** † Department of Chemistry and Centre for Processable Electronics, Imperial College London, London W120BZ, United Kingdom; ‡ KAUST Solar Centre (KSC), King Abdullah University of Science and Technology (KAUST), Thuwal 23955−6900, Saudi Arabia

## Abstract

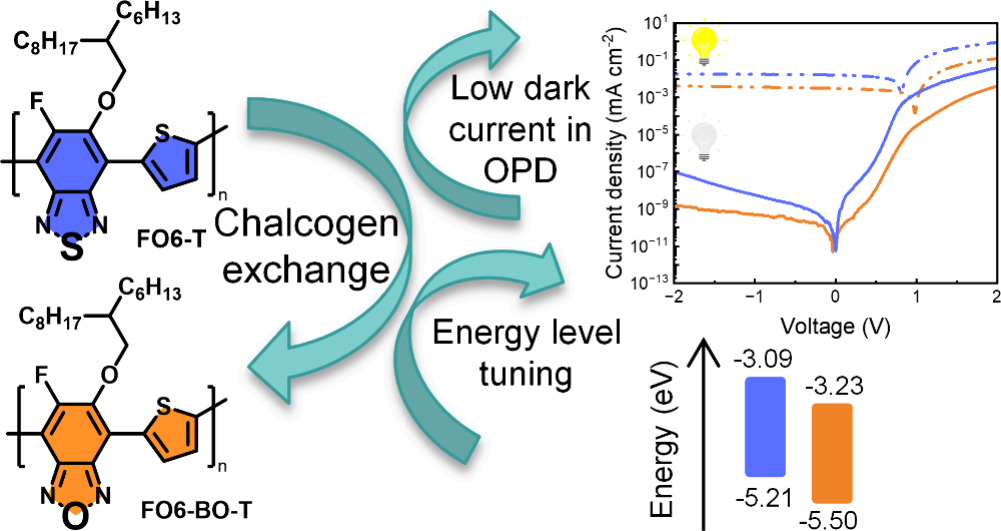

The performance of organic photodetectors (OPDs) using conjugated polymer donors and molecular acceptors has improved rapidly, but many polymers are difficult to upscale due to their complex structures. This study examines two low-complexity thiophene copolymers with substituted benzooxadiazole (**FO6-BO-T**) or benzothiadiazole (**FO6-T**). Substituting sulfur with oxygen in **FO6-BO-T** increased its ionization energy without affecting the optical gap. When blended with the nonfullerene acceptor IDSe, **FO6-BO-T** showed a significantly lower dark current density (2.06·10^–9^ A cm^–2^ at −2 V) compared to **FO6-T**. Grazing incidence wide-angle X-ray scattering (GIWAXS) measurements demonstrated that pristine **FO6-BO-T** exhibited a more ordered morphology than **FO6-T**. However, blending resulted in a significant disruption to the ordered domains in both cases, with a loss of orientational order, suggesting that **FO6-BO-T**’s improved performance is largely related to its increased ionization energy. This study demonstrates the potential of chalcogen atom engineering to enhance the performance of the OPD in scalable polymers.

Photodetectors, devices designed to convert incoming light into electrical signals, hold a pivotal role in modern technology and are central to numerous applications, ranging from telecommunications and imaging technologies to environmental monitoring and machine vision.^1−4^ The global demand for photodetectors continues to surge, driven by the ever-expanding need for faster, more sensitive, and energy-efficient devices in an increasingly interconnected world.^5^ Traditional photodetector materials, such as GaAs, have long dominated the market due to their excellent performance characteristics. They are known for their high speed and sensitivity, as well as excellent quantum efficiency.^6^ However, traditional photodetectors based on GaAs and similar materials exhibit certain shortcomings. Their manufacturing involves intricate and resource-intensive processes, leading to elevated production costs.^7^ This cost factor can be a significant hindrance, particularly in applications requiring large-scale deployment.

Organic photodetectors (OPDs) exhibit significant potential advantages over their inorganic counterparts in terms of large-area manufacturing, as they are suitable for solution processing and roll-to-roll printing techniques.^4,8^ Moreover, they offer broad and tunable absorption as well as the option for flexible device preparation which makes them superb candidates for large-area flexible imagers, on-the-go monitoring, and skin-grafted sensors and bioelectronics.^9−12^ Typically OPDs are fabricated from a blend of electron donor and electron acceptor materials, with the donor often being polymeric and the acceptor a molecular material.^4,6,13,14^ Such a blend, termed a bulk heterojunction (BHJ), maximizes the interfacial area between the two materials, which is important for maximizing light to current conversion via exciton harvesting.^15^

An area of significant interest has been the development of near-infrared (NIR) OPDs.^16−18^ Here a focus has been on improving the responsivity (*R*) and specific detectivity (D*) of devices. This can be limited in NIR devices due to high nonradiative recombination in narrow band gap materials, which results in poor exciton dissociation efficiency.^19,20^ This is typically overcome by operating the device with a negative external bias, which helps exciton dissociation but results in increased dark current density (*J*_*D*_) due to charge injection from the electrode.^21−25^ However, there is a playoff since *R* and D* are dependent on both high photocurrent and low dark current densities. Dark current density can depend on a variety of factors, such as BHJ morphology, energetic disorder, and charge recombination processes. The chemical structure of the two blend materials is also critically important and therefore the design of new materials exhibiting low dark current is an important area for further research.^11,24,26−29^

The performance of OPD devices has seen significant progress with a variety of blends now reported which exhibit high photocurrent and low dark current densities.^30−34^ However, the focus on improving device performance has arguably come at the expense of synthetic complexity, with many of the donor polymers utilized in OPD’s requiring complex, multistep synthesis. This potentially negatively impacts the scalability and cost. Recently we published a protocol for the preparation of efficient donor polymers for organic photovoltaic (OPV) devices, which were prepared in just two synthetic steps from a readily accessible building block based on benzo[*c*][1,2,5]thiadiazole (BT).^35^ We identified a promising donor material, **FO6-T**, based on a copolymer of BT and thiophene, which was readily scalable and exhibited good device stability under illumination. Given the simplicity of **FO6-T** we were interested in studying its performance in the OPD devices, and here we report the outcome of those studies. However, one limitation of **FO6-T** was its relatively small ionization energy (I.E.), which limited the available solar cell open circuit voltage (*V*_*OC*_) available. Several studies have highlighted the relationship between dark current in OPD blends and the *V*_*OC*_ in OPV blends.^9,19^ Therefore, in addition to investigating **FO6-T**, we also aimed to develop an analogue exhibiting a higher I.E.

In order to increase the I.E. of **FO6-T**, we focused on exchanging the sulfur atom in the BT for oxygen. Although BT is widely used as an electron accepting unit in conjugated materials, materials based on its oxygen analogue, benzo[c][1,2,5]oxadiazole (BO) remain much rarer.^36−42^ Oxygen is more electronegative than sulfur, making the BO monomer more electron accepting, which was expected to make the resulting polymer harder to oxidize. Increasing I.E. is known to strongly correlate to the *V*_*OC*_ which can be obtained in photovoltaic devices.^43^ On this basis, we hereby report a novel conjugated polymer, **FO6-BO-T** which was synthesized by a two-step protocol starting from a fluorinated BO precursor. The influence of the change in heteroatom was studied in terms of optical and electronic properties, as well as their OPD device performance. In the OPD devices, when blended with a nonfullerene acceptor material, the BO based device exhibits a notably lower dark current than the BT analogue, with a *J_d_* of 2.06·10^–9^ A cm^–2^ together with a 3.17·10^11^ Jones specific detectivity (D*) value at 780 nm under −2 V bias. The differences between the two polymer blends were also investigated in terms of their thermal properties and thin-film microstructure.

Interestingly, our findings show, that compared to organic photovoltaic (OPV) devices,^44^ the energy alignment of the donor and acceptor materials seems to be the major contributor to the change in device parameters. These findings highlight the utility of molecular design and minor modifications to the molecular structure as an effective method for fine-tuning the active layer of an OPD device. We furthermore emphasize the importance of shifting the focus to low synthetic complexity materials, especially with large-scale applications in mind.

Our recent synthesis of **FO6-T** utilized a nucleophilic aromatic substitution (S_N_Ar) reaction with an alcohol on readily available 4,7-dibromo-5,6-difluorobenzo[*c*][1,2,5]thiadiazole, forming the monofluorinated monomer in good yield. To the best of our knowledge, the equivalent S_N_Ar reaction on 4,7-dibromo-5,6-difluorobenzo[*c*][1,2,5]oxadiazole has not been reported. The starting 4,7-dibromo-5,6-difluorobenzo[*c*][1,2,5]oxadiazole is commercially available, but can also be prepared from readily available 3,4-difluoro-5-nitroaniline in four steps.^45^ Reaction with one equivalent of 2-hexyl-1-decanol in the presence of NaH in THF, afforded the monomer **FO6-BO** in a yield of 35% after chromatographic purification (Scheme 1). The structure of the monomer was confirmed by a combination of NMR and mass spectroscopy (see SI). We note that the yield is lower than the BT analogue, but no modifications to the synthesis were explored to improve the yield or purification at this stage. Subsequently, the monomer **FO6-BO** was copolymerised following a Stille protocol with 2,5-bis(trimethylstannyl)thiophene (**T**).^46^ After Soxhlet extraction of the resulting polymer using methanol, acetone, hexane, and chloroform (in that order), the polymer was isolated from the chloroform fraction. **FO6-BO-T** showed good solubility in chloroform and was obtained in a yield of 98%. **FO6-T** was prepared following the published protocol and was obtained in a high yield of 92%.

**Scheme 1:**
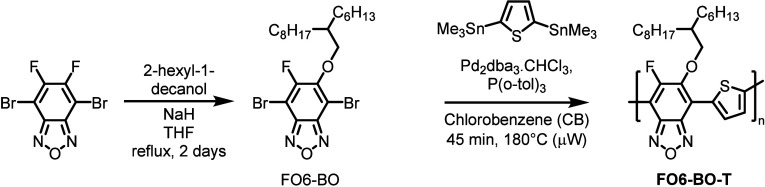
A Two-Step Synthetic Protocol: Nucleophilic Aromatic Substitution Followed by Stille Polymerization under Microwave Conditions

The molecular weights of the obtained polymers were initially measured by gel permeation chromatography (GPC) in chlorobenzene at 80 °C, as we previously used for **FO6-T.** However, **FO6-BO-T** exhibited a very high tendency to aggregate under these conditions, and the molecular weight could not be determined. Moving to high temperature GPC with 1,2,4-trichlorobenzene at 150 °C against poly(styrene) standards helped. For **FO6-BO-T** a *M*_*n*_ of 15 kDa (*M*_*w*_ = 68 kDa) with a *Đ* of 4.6 was obtained, and for **FO6-T** a *M*_*n*_ of 24 kDa (*M*_*w*_ = 69 kDa) and a *Đ* of 2.9 was obtained (see Figure S6).

The optical properties of the novel polymer **FO6-BO-T** were determined using UV–vis absorption and photoluminescence (PL) spectroscopy and compared to the properties of **FO6-T**, as shown in Figure 1 and Figure S7. The spectra for both polymers are very similar, in solution and in thin film, with the 0–0 and 0–1 peaks of a vibronic progression most clearly observed in the film but also apparent in solution. We previously ascribed these features to the good planarity of the polymer backbone, possibly as a result of intramolecular noncovalent interactions, since they did not exhibit any significant change on heating.^35^ There is also very little shift on moving from solution to film, with the only change being in the relative intensities of the 0–0 and 0–1 peaks. The peak maxima are summarized in Table 1, and it is apparent that changing the heteroatom results in very little change. Similarly, the optical absorption onsets are almost identical, in both solution and film, corresponding to optical band gaps (*E_g,opt_*) around 1.69 eV (see Table 1).

**Table 1:** Optical Characterization of the Polymers in Solution and Thin Film Determined by UV–vis and PL Spectroscopy

	*λ_max,abs_* (CHCl_3_) (nm)	*λ_max,abs_* (film) (nm)	*λ_max,PL_* (nm)	*E_g,opt_* (eV)^a^
**FO6-T**	625, 650 (sh)	619, 665	713	1.69
**FO6-BO-T**	624, 665 (sh)	619, 669	740	1.69

a Estimated from the onset of absorption from the measured thin-film UV–vis spectra where *E* = *hc*/λ

**Figure 1:**
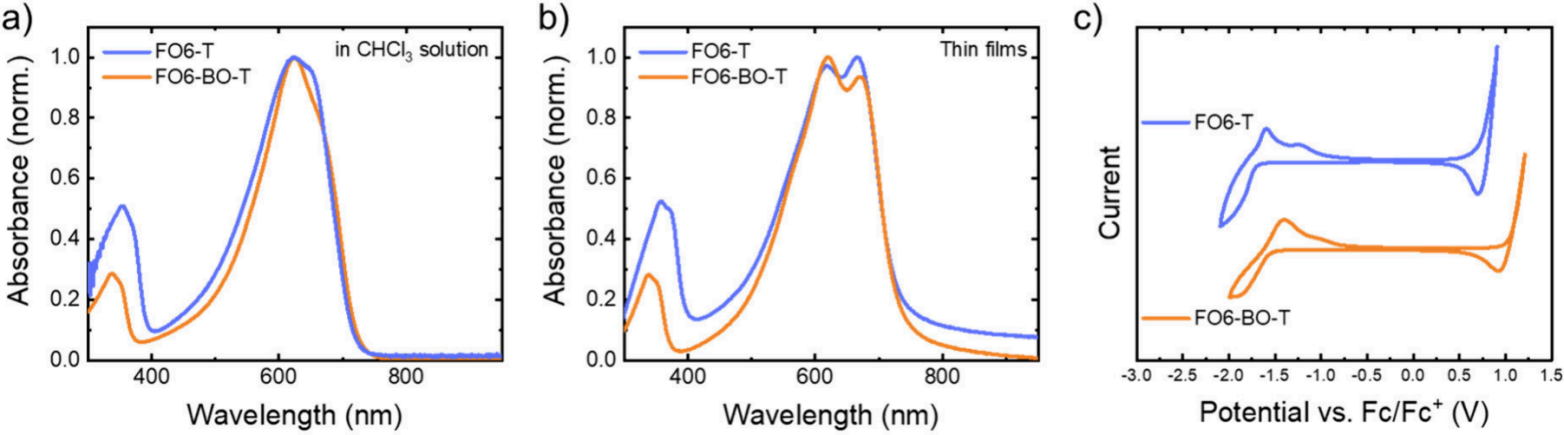
a) UV–vis spectra of solutions of polymers in CHCl_3_, b) UV–vis spectra of spin-coated thin films, and c) cyclic voltammograms of drop casted thin films. The voltammograms were recorded using a glassy carbon working electrode on which the films were drop-casted, a platinum counter electrode, and a silver wire quasireference electrode (QRE) at a scan rate of 0.1 V s^–1^. 0.1 M [*n*-Bu_4_N]PF_6_ in MeCN was used as the supporting electrolyte.

To investigate the impact of the different heteroatoms, the electronic properties of thin films were investigated using a combination of techniques. Thus, photoelectron spectroscopy in air (PESA) and ultraviolet photoelectron spectroscopy (UPS) were utilized to measure the I.E, in ambient and vacuum respectively, while the electron affinity (E.A.) was measured with low energy inverse photoemission spectroscopy (LE-IPES). These were complemented with oxidation and reduction potential measurements by cyclic voltammetry (CV) in the presence of a solution electrolyte, referenced against ferrocene. The results are summarized in Table 2. The experimental measurements were supported by theoretical calculations derived from density functional theory (DFT), employing the B3LY/6.31G (d,p) level of theory to calculate the highest occupied molecular orbital (HOMO) and lowest unoccupied molecular orbital (LUMO) values. In these computations, the 2-hexyl-1-decyl groups were substituted with isopentyl groups and the polymers were approximated as trimers for computational efficiency (see Figure S8).

**Table 2:** Summarized Results of Electronic Properties of the Polymers Determined by CV, PESA, UPS, and LE-IPES

**Polymer**	**CV**^a^			**PESA**^b^	**UPS/LE-IPES**			**DFT**	
	*E*_*ox*_ (eV)	*E_red_* (eV)	*E_g,CV_* (eV)	I.E. (eV)	I.E. (eV)	E.A. (eV)	*E_fund_* (eV)	HOMO (eV)	LUMO (eV)
**FO6-T**	–5.51	–3.09	2.42	–5.21	–5.10	–3.04	2.06	–5.06	–2.84
**FO6-BO-T**	–5.82	–3.23	2.59	–5.50	–5.25	–3.45	1.80	–5.33	–3.08

a Energy levels were estimated from cyclic voltammetry onset potentials for oxidation and reductions peaks, using a Fc/Fc^+^ standard of −4.8 eV, in the solid state with a Ag/Ag+ reference electrode at a scan rate of 0.1 V s^–1^ and tetrabutylammonium hexafluorophosphate in acetonitrile (0.1 M) as the supporting electrolyte.

b PESA measurements were performed in thin films spin-coated on ITO. The error of both techniques is around ±0.1 eV.

The general trend of all measurements is clear from Table 1, that changing from **FO6-T** to **FO6-BO-T** results in a reduction in both the I.E./HOMO and E.A/LUMO energy levels, as hypothesized due to the incorporation of the more electronegative oxygen compared to sulfur. The absolute value for the energy levels varies according to technique, and it is difficult to compare between techniques because of the very different conditions and relative errors, as recently noted.^47^ Nevertheless all techniques agree that the HOMO deepens by approximately 0.15–0.3 eV upon changing from BO to BT. This value is further supported by the DFT calculations, which predict a change of 0.27 eV.

There is a larger variance in the LUMO shift with CV giving a deepening of 0.14 eV (in *E_red_*). This results in a wider electrochemical bandgap for **FO6-BO-T** compared to **FO6-T**, although the optical band gaps are identical. On the other hand, LE-IPES suggests a deepening in the E.A. of 0.41 eV. The difference between the UPS and LE-IPES values can be used to determine the fundamental gap *E_fund_*. In this case, it is smaller for **FO6-BO-T** compared to **FO6-T.** Again, given the identical optical band gap, this would suggest that the exciton binding energy **E_b_** is reduced on changing from BT to BO. This would be surprising given the similar optical gap and chemical structure, and we believe it is related to the error associated with the LE-IPES measurements and associated data fitting (see Figure S9). In summary, all experimental and theoretical results point to a deepening of both HOMO and LUMO upon a heteroatom change from S to O.

To determine the degradation (*T*_*d*_) and glass transition temperatures (*T*_*g*_) for the polymers, thermogravimetric analysis (TGA) as well as differential scanning calorimetry (DSC) techniques were used. Furthermore, due to the inaccessibility of the *T*_*g*_ via DSC, an optical method using changes in UV–vis spectroscopy of heated thin-films of the materials was employed.^48^ The degradation temperatures were determined by TGA, with the curves shown in Figure S10. The scans were heated from 25 to 750 °C at 5 °C/min under N_2._ Both polymers show high thermal stability, with a *T*_*d*_ (5% weight loss) for **FO6-BO-T** of 307 °C and for **FO6-T** a *T*_*d*_ of 305 °C. DSC analysis of the polymers is shown in Figure S11, but did not reveal any features for either polymer. For many conjugated polymers, the *T*_*g*_ is too weak to be detected by DSC.^48^

To get some information about the temperature associated with morphological changes in the bulk material, we used a method described by Lipomi and colleagues that tracks molecular-scale rearrangements in the thin film upon heating, which are monitored through changes in the UV–vis spectrum.^48^ Thus, spun-cast thin films of the polymers were heated in 10 °C increments using a film heating set up. After the temperature stabilized and following a 2 min annealing period, the UV–vis spectrum was recorded. The normalized absorption spectra of the respective polymers at temperatures ranging from room temperature to 170 °C are shown in Figures S12a and S13a. As reported, we applied a deviation metric DM_T_ describing the sum of the changes of the absorbance between the as-cast polymer film and the annealed film at temperature *T* (see SI).^48^ In order to determine the *T*_*g*_ for each polymer, the DM_T_ was plotted against the annealing temperature, and the change in slope was determined by intersecting between the two fit lines. In Figures S12b and S13b, the DM_T_ values for the polymers are plotted versus annealing temperature. For **FO6-BO-T**, the change of the slope and hence the *T*_*g*_ of the polymer was determined to be at 98 °C. For **FO6-T**, a distinct change in slope can be observed and the *T*_*g*_ was estimated to be slightly higher at 106 °C. We note the technique only gives an indication of where the *T*_*g*_ is located but nevertheless suggests a reduction upon changing from S to O.

The polymers were tested as donor materials in OPDs in combination with a recently reported nonfullerene acceptor material, IDSe, which exhibits a complementary absorption to the near-IR region and energetics to form a type II offset with the donor polymers.^49^ The molecular structures and energy levels are shown in Figure 2a, and the absorption spectra of IDSe and the polymers are shown in Figure 2b. The OPDs were fabricated with an inverted architecture, based on ITO/ZnO/Active layer/MoO_3_/Ag (see Figure 2c), using a 1:1.5 ratio (wt%) of donor to acceptor. Devices were annealed at 100 °C for 10 min prior to deposition of the MoO_3_/Ag.

**Figure 2:**
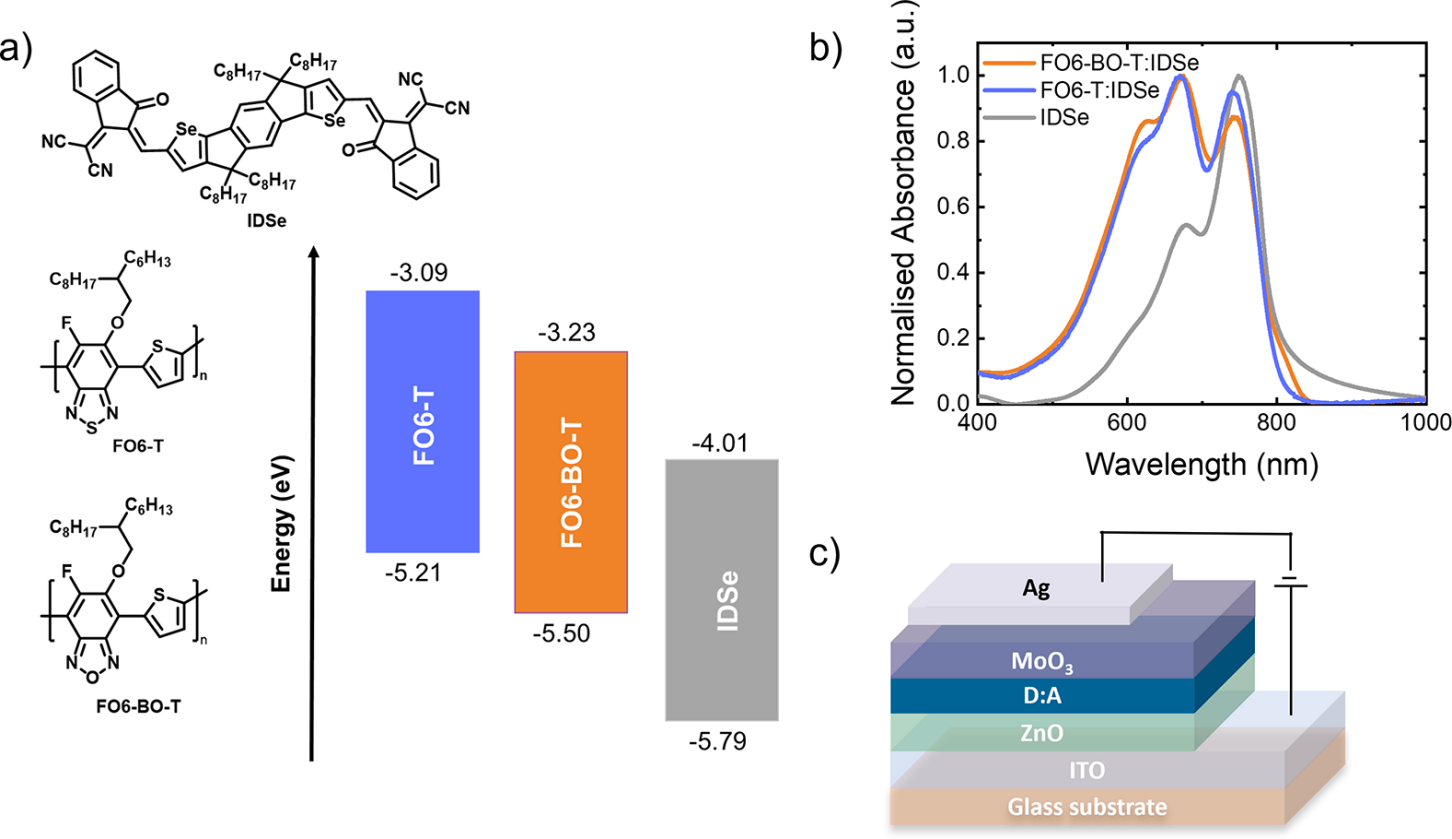
a) Chemical structures of the donors **FO6-T** and **FO6-BO-T** and the acceptor IDSe and their energy diagram. Energy levels determined by PESA (HOMO) and CV (LUMO) are depicted. b) Normalized thin film absorption of the pristine IDSe as well as D/A blends. c) Schematic of the OPD device with inverted architecture.

The current density–voltage (*J*–*V*) characteristics as measured under one sun (AM1.5G) illumination and in the absence of light conditions are shown in Figure 3a. We found that devices based on **FO6-BO-T:**IDSe system exhibited very low dark current density of 2.06·10^–9^ A cm^–2^ at −2 V, which is among the lowest *J_d_* values reported in the literature for OPDs with detection above 750 nm.^5,6,9^ If compared to **FO6-T**:IDSe, a higher *J_d_* of 9.07·10^–8^ A cm^–2^ was obtained under the same bias. **FO6-BO-T** devices exhibit a higher *V*_*OC*_ than **FO6-T** under illumination, as expected from the I.E. measurements, which further validates the correlation between dark current and *V*_*OC*_.^19^ All the OPD characterization data are summarized in Table 3. To achieve effective photodetection in OPD devices, it is crucial to ensure the efficient conversion of photons into electrons. Responsivity (*R*), which is closely associated with the external quantum efficiency (EQE), can be calculated from the following equation,
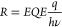
1

**Figure 3:**
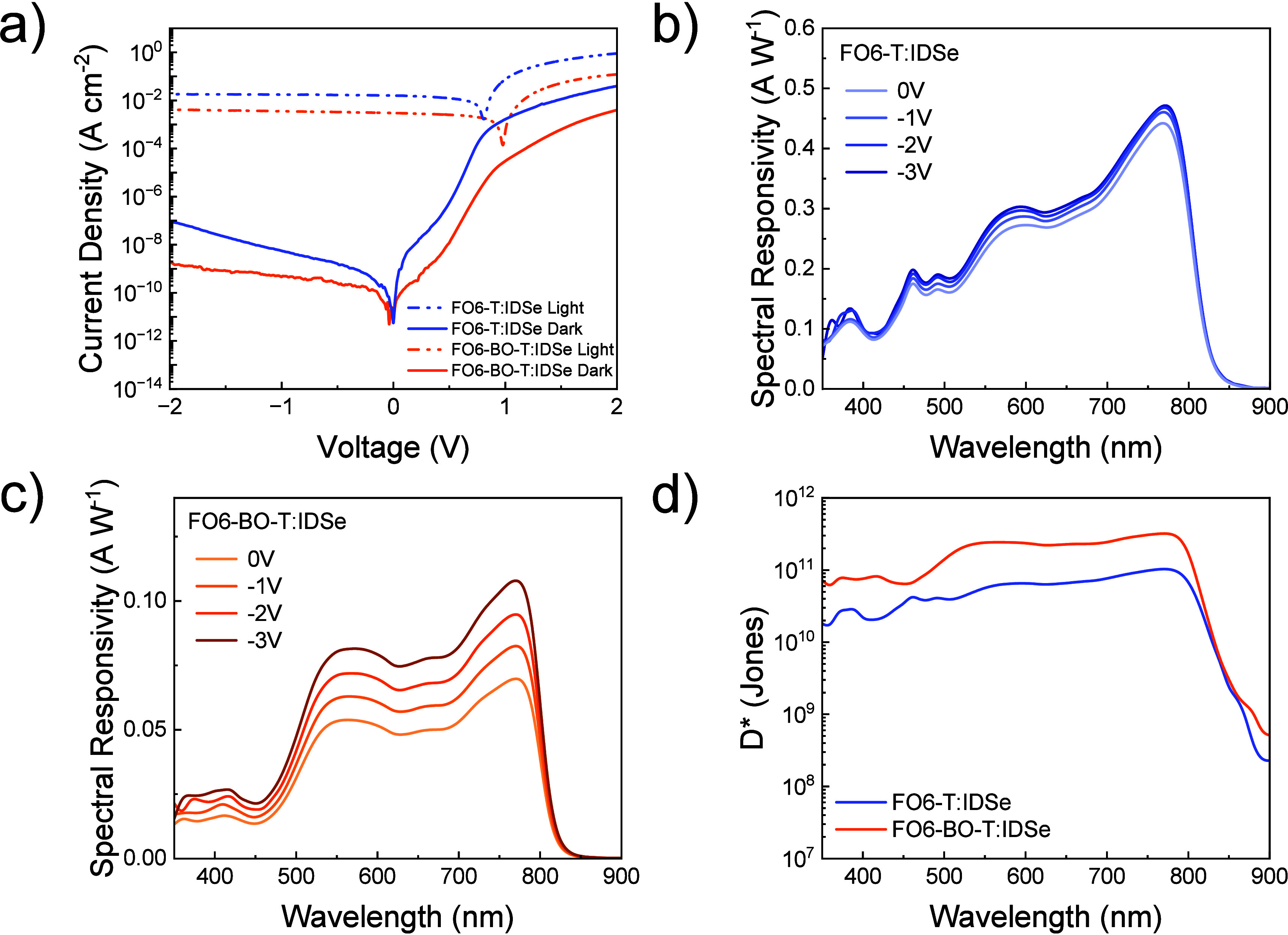
a) Current density–voltage characteristics of **FO6-T**:IDSe and **FO6-BO-T**:IDSe OPDs under AM1.5G illumination and in dark conditions, b) and c) responsivities, and d) specific detectivity (D*) of the OPD devices under −2 V.

**Table 3:** Key Performance Parameters of the OPDs Based on **FO6-T** and **FO6-BO-T** Blended with Acceptor IDSe^a^

Blend	*J_d_* (A cm^–2^)	*R* (A W^–1^)	D* (Jones)	Cut-off frequency (kHz)	LDR (dB)
**FO6-T:IDSe**	9.07·10^–8^	0.45	1.02·10^11^	375	102
**FO6-BO-T:IDSe**	2.06·10^–9^	0.09	3.17·10^11^	312	126

a Dark current density (*J_d_*), responsivity (*R*), LDR and specific detectivity (D*). All were reported at 780 nm and −2 V reverse bias.

R plots for **FO6-T** and **FO6-BO-T** blends with IDSe are shown in Figure 3b and c respectively, at different reverse biases, ranging from 0 to −3 V. For both blends, a bias dependency of the responsivity can be observed, which can be associated to enhanced charge extraction capabilities of these blends at reverse bias.^50^ The difference in responsivity between **FO6-T**:IDSe and **FO6-BO-T**:IDSe devices might arise from the lower HOMO–HOMO offset between **FO6-BO-T** and IDSe compared to those between **FO6-T** and IDSe. This is evident by the strongest bias-dependent responsivity in FO6-BO-T:IDSe devices.^50^ Another key figure of merit for OPDs is the specific detectivity D*, which can be derived from equation 2.
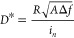
2Where *A* refers to the photoactive pixel area, *Δf* is the detection bandwidth and *i*_*n*_ is the noise current spectral density.^3^ Apart from shot noise, which is nominated by **J_d_**, flicker and thermal noise will also make substantial contributions to the overall noise in the device.^51^ Therefore, we obtained the noise current spectral density (Figure S14a) through a Fast Fourier Transform (FFT) of the noise current recorded using a digital oscilloscope to avoid overestimating the D* value.^26^ Specific detectivity was determined to be 3.17·10^11^ Jones at −2 V for the **FO6-BO-T** system (see Figure 3d), at 780 nm. In comparison, the **FO6-T** blend exhibits reduced performance, with a lower D* of 1.02·10^11^ Jones under the same operating condition, in accordance with the trends observed for the *J_d_*. We note that D* was calculated using the measured noise and under −2 V of reverse bias, following the recommended protocol.^51^ Higher values can be obtained by measuring at lower reverse bias or using calculated noise, and many literature reports use this method. For comparative purposes, we note that the values of D* using the calculated noise are significantly higher at 3.36·10^12^ Jones for the **FO6-BO-T** blend (see SI, Figure S14 and S15).

Apart from the ability to detect low light levels, a photodetector with a linear response to varying light intensities is particularly valuable, especially in indoor applications or high-resolution image sensors.^52^ This can be quantified by linear dynamic range (LDR) using equation 3;
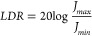
3where *J*_*max*_ and *J*_*min*_ represent the upper and lower linearity limits. LDR was measured to be 126 dB at 780 nm for the blend with **FO6-BO-T** (Figure 4b) and 102 dB for **FO6-T** blends under −2 V bias (Figure 4a). In order to match the requirements for fast imaging, video, and communication applications, minimum detection speed demands need to be met.^10^ Hence, we performed dynamic characterization on the OPDs, such as cutoff frequency (Figure 4c) as well as the rise and fall times (*t_r_* and *t_f_*) (Figure 4d). Cut-off frequency, limited by the charge carrier transient time, refers to the frequency where the device’s photocurrent response drops to 70% of its original value (−3 dB). The **FO6-BO-T**: IDSe blend features a cutoff frequency of 312 kHz at 780 nm and −2 V, well above the minimum required for video applications. The rise time describes the time-interval for the signal to respond between 10% and 90% of the maximum value and vice versa for fall time. For **FO6-BO-T**:IDSe *t_r_* was identified to be 1.65 μs, and the *t_f_* 1.41 μs. Interestingly the **FO6-T**:IDSe device exhibits significantly improved dynamic properties, with a high cutoff frequency of 375 kHz, as well as a *t_r_* of 0.31 μs and *t_f_* of 0.23 μs. We correlated the dynamic characterization of the asymmetric polarized OPDs with the space-charge-limited current (SCLC) mobilities (see Table S1 and Figure S16). **FO6-T**:IDSe blends show high electron and hole mobilities of 3.11·10^–5^ and 5.60·10^–5^ cm^2^/(V s) respectively. **FO6-BO-T**:IDSe exhibits slightly lower electron and hole mobilities of 8.66·10^–6^ and 4.40·10^–6^ cm^2^/(V s), which are in accordance with the slower rise and fall times of this blend.

**Figure 4:**
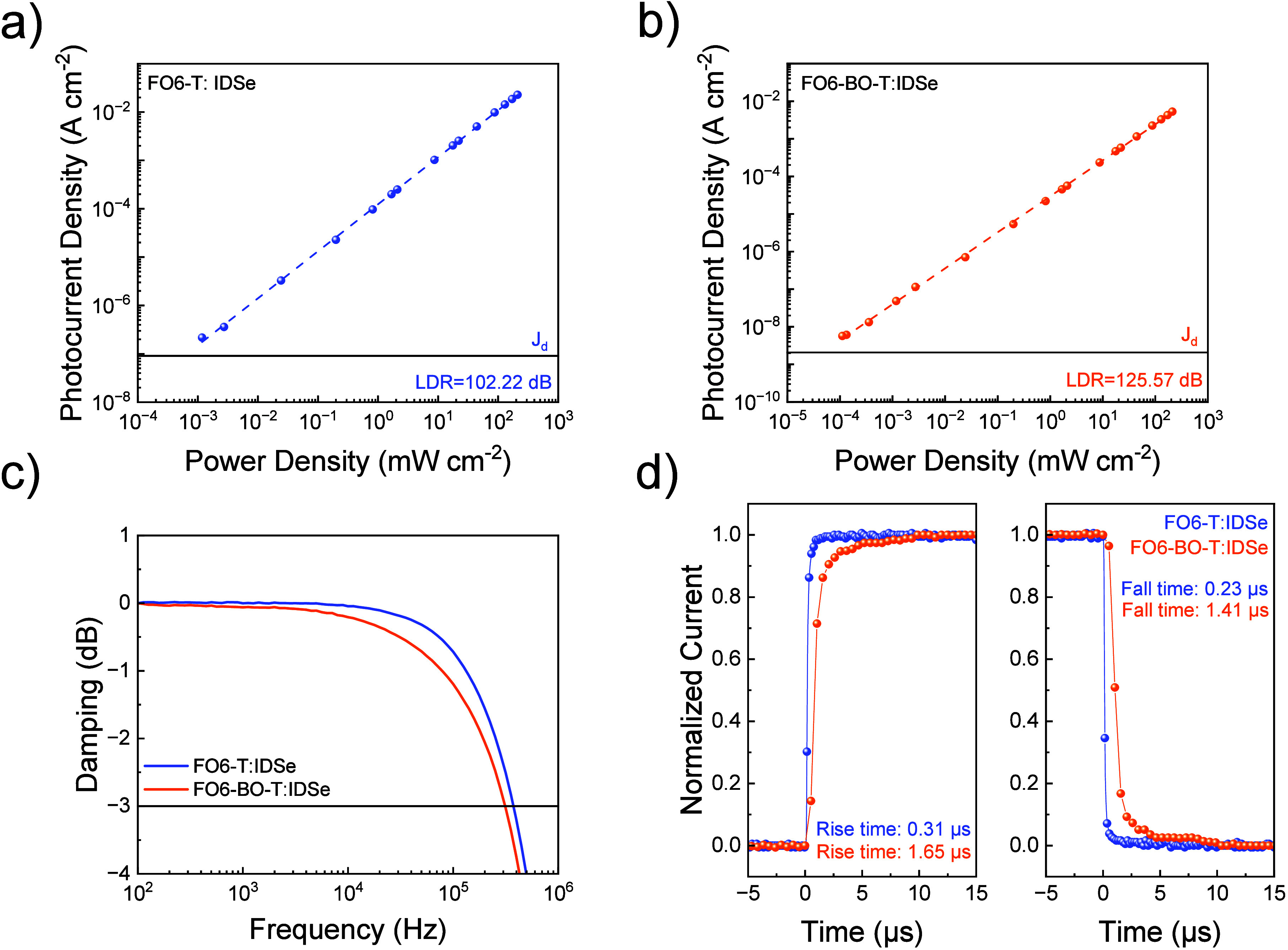
LDR measurements at −2 V and under white light illumination of a) **FO6-T**:IDSe and b) **FO6-BO-T**:IDSe, c) cutoff frequency at −2 V and 780 nm and d) transient photocurrent measurements with rise and fall times for **FO6-T**:IDSe and **FO6-BO-T**:IDSe.

To elucidate the effect of the heteroatom on film morphology, grazing-incidence wide-angle X-ray scattering (GIWAXS) was measured on pristine polymer films as well as their blends with the IDSe acceptor (see Figure 5 and Table S2). Pristine **FO6-T** exhibited predominant face-on orientation as seen through an out-of-plane π–π [010] diffraction peak and in-plane lamellar [100] and [200] peaks. Weakly visible out-of-plane lamellar peaks suggest some degree of edge-on orientation as well, although to a lesser extent. Q-values of 1.61 and 0.28 Å^–1^ for π–π and lamellar peaks respectively (corresponding to *d*-spacings of 3.88 and 22.4 Å) agree well with our previous reports, although the use of benchtop equipment resulted in lower peak intensities than previous measurements conducted at synchrotron facilities.^35^ Meanwhile, **FO6-BO-T** exhibited edge-on orientation, with 4 orders of out-of-plane lamellar diffraction peaks, indicating a more ordered morphology compared to **FO6-T**. The [100] q-value of 0.30 Å^–1^, corresponds to a *d*-spacing of 20.9 Å, which is slightly shorter than that of **FO6-T**. A very weak π–π peak can be discerned in the line-cut at q = 1.65 Å^–1^ (Figure 5f), also corresponding to a slightly shorter *d*-spacing of 3.81 Å. It is noteworthy that both polymers exhibit well-ordered films, despite the lack of apparent regiocontrol in the polymerization. This is in agreement with their high tendency to aggregate and planarize in solution. Assessment of regioregularity by ^1^H or ^19^F NMR is complicated by the poor resolution of the spectra, even at high temperature (see SI, Figures S4 and S5), which relates to the high tendency to aggregate. Studies on preformed monomers are in progress to try to precisely control the regioregularity.

**Figure 5:**
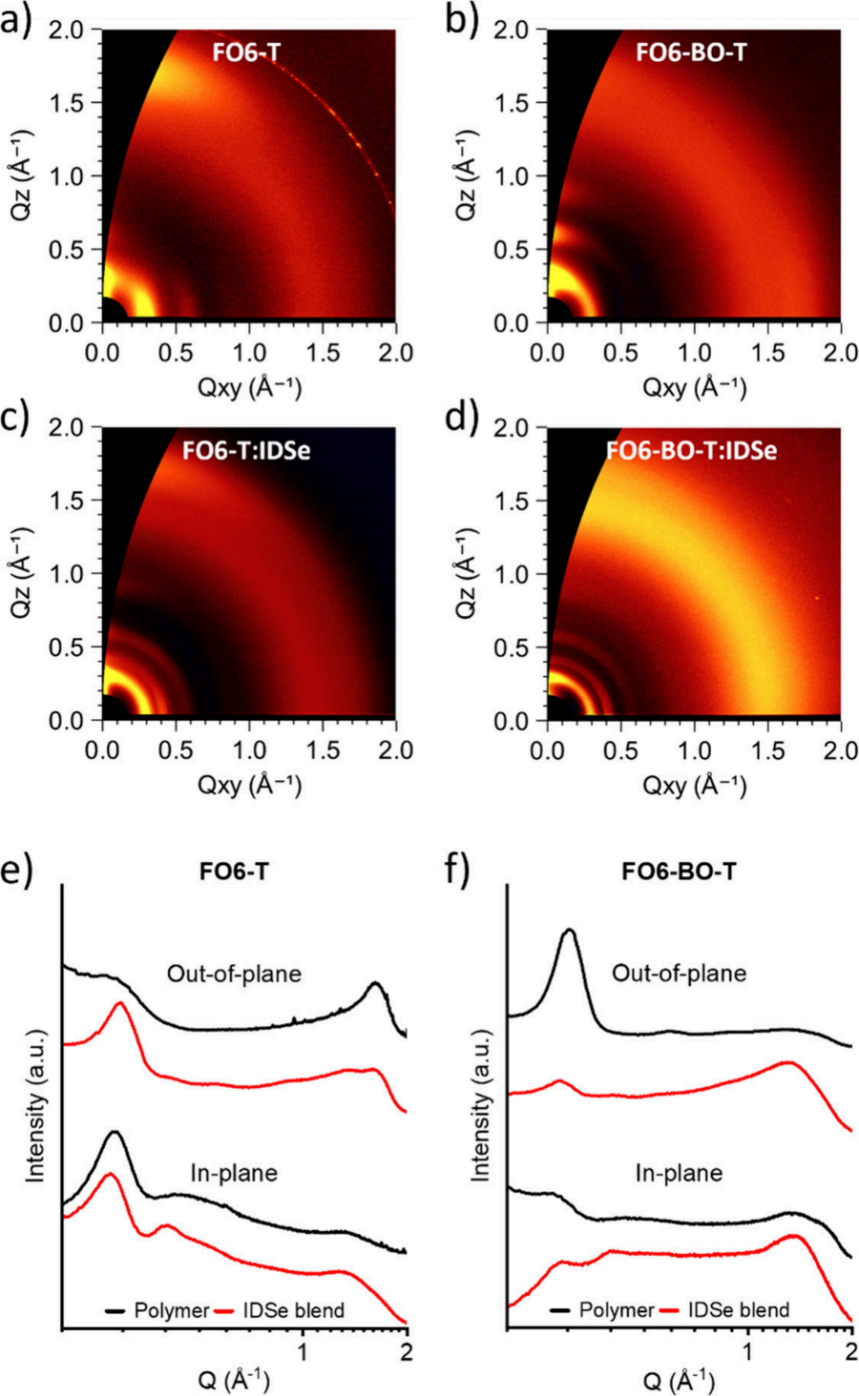
2D GIWAXS images (a-d) of the polymers and their blends with IDSe and (e,f) 1D line plots of the polymers and their blends.

Blending with IDSe results in a significant disruption to the ordering observed in pristine films (Figure 5c,d). Peaks corresponding to polymer (100) are observed in both cases, together with those attributable to the IDSe, suggesting that crystalline regions of both materials are present. However, there is a loss of order within crystallites, particularly with respect to highly ordered pristine IDSe (Figure S17),and orientational preference is lost, with blends appearing largely isotropic. Microstructure plays an important role in device performance,^53−55^ and a more pronounced face-on morphology could help to explain the improved hole mobility in the out-of-plane direction. While this may be a contributing factor to the different device performance, the fact that both blends appear largely isotropic leads us to conclude that the improved performance of the **FO6-BO-T**:IDSe blend is mainly correlated to the improved energy alignment between donor and acceptor.

In conclusion, a novel polymer, namely **FO6-BO-T** was synthesized using a two-step method incorporating the relatively unexplored acceptor unit benzo[*c*][1,2,5]oxadiazole. The polymer properties were compared to those of **FO6-T**, to study the effect of varying the chalcogen in the acceptor unit of the polymer. By incorporation of the more electronegative oxygen instead of sulfur, the ionization energy of the polymer was increased, but the optical band gap was unchanged. The changes in energetic levels were confirmed by a variety of characterization methods such as CV, PESA, UPS and LE-IPES, as well as computationally using DFT, clearly demonstrating that the electronic properties of the polymer can be controlled via small changes of the structure. Blends of both polymers with the nonfullerene acceptor IDSe were investigated in OPD devices with an inverted device architecture. Specific detectivity (D*) values were calculated at a reverse bias of −2 V, using measured values of the noise current spectral density to avoid overestimation, with **FO6-BO-T** exhibiting a value of 3.17·10^11^ Jones at 780 nm, a 4-fold improvement over **FO6-T** (values of D* using calculated noise are 1 order of magnitude higher). These differences were mainly related to the significantly lower dark current for **FO6-BO-T**, 2.06·10^–9^ A cm^–2^ at −2 V versus 9.07·10^–8^ A cm^–2^ for **FO6-T**. Interestingly, **FO6-T** devices exhibited better dynamic properties, with a high cutoff frequency of 375 kHz, which was related to the higher hole and electron mobilities in this blend compared to **FO6-BO-T** containing blends. Examination of the polymer microstructure by GIWAXS demonstrated that pristine **FO6-BO-T** exhibited a more ordered morphology than that of **FO6-T**, with a predominant edge-on orientation. However, blending resulted in a significant disruption to the ordered domains in both cases, with a loss of orientational order. Thus, we conclude, that the improved performance of **FO6-BO-T** in OPDs can be attributed primarily to the improved energy alignment of donor and acceptor compared to devices based on **FO6-T**. Overall, chalcogen engineering is demonstrated to be a useful strategy for preparing a low-complexity donor polymer for highly efficient OPDs, and we believe that this is especially interesting with regard to large scale applications.

## Supporting Information Available

General information, experimental procedures and analytical data (^1^H, ^19^F, and ^13^C NMR spectra), GPC traces, PL spectra, DFT calculations, UPS and LE-IPES spectra, TGA and DSC traces, temperature dependent UV/vis spectra and deviation metrics, device fabrication details, JV curves, *D** plots, GIWAXS plots (PDF)
