# Supplementary material for: Energy Level Tuning in Conjugated Donor Polymers by Chalcogen Exchange for Low Dark Current Organic Photodetectors
Source: ACS Mater Lett. 2024 Oct 8;6(11):5006–15. doi: 10.1021/acsmaterialslett.4c01899 (PMC11539101; doi:10.1021/acsmaterialslett.4c01899)
Supplement: Supplementary file 1 — tz4c01899_si_001.pdf [file tz4c01899_si_001.pdf]

Supporting Information

Energy level tuning in conjugated donor polymers by chalcogen exchange for low dark current organic photodetectors

Martina Rimmele,[a] Zhuoran Qiao,[a] Filip Aniés, Adam V. Marsh,[b] Aren Yazmaciyan,[b†] George Harrison,[b] Shadi Fatayer,[b] Nicola Gasparini,*[a] Martin Heeney*[a,b]

[a] Department of Chemistry and Centre for Processable Electronics, Imperial College London, London, W120BZ, United Kingdom

[b] KAUST Solar Centre (KSC), King Abdullah University of Science and Technology (KAUST), Thuwal, 23955–6900, Saudi Arabia

[†] Current address: Paul-Drude-Institut für Festkörperelektronik, Leibniz-Institut im Forschungsverbund 10117 Berlin e.V., 10117 Berlin, Germany

Contents

1. Methods 2

2. Experimental 5

3. NMR Spectra 7

4. Molecular weight characterisation 9

5. Photoluminescence 10

6. DFT 110

7. UPS/LE-IPES 10

8. Thermal Characterisation 11

9. Device Fabrication 13
10. OPD Characterisation 14
11. Charge carrier mobilities 15
12. GIWAXS 17
13. References 18

1. Methods

All chemicals were used as received and purchased from major chemical suppliers. Synthetic reactions were carried out under nitrogen atmosphere using standard Schlenk techniques. Characterisation was carried out in air at room temperature unless stated otherwise.

^{1}H NMR, ^{19}F NMR and ^{13}C NMR were collected either on AV-400 (MHz) or AV-500 (500 MHz) Bruker Spectrometer using *d*-chloroform as solvent.

Recycling preparative GPC was performed with a LaboACE LC-5060 equipped with Jaigel 2HR and 2.5HR columns, 20 mm ID × 600 mm each.

Polymers were purified prior to device fabrication on a Shimadzu **GPC** system running in chloroform at 40°C with an Agilent PLgel 10 µm mixed-d column, DGU-20A3 Degasser, LC-20A Pump, CTO-20A Column Oven and SPD-20A UV Detector.

UV-vis absorption spectra were recorded on an Agilent Cary 60 UV-vis spectrophotometer at room temperature.

Photoluminescence (PL) spectra were acquired on an Agilent Cary Eclipse fluorescence spectrophotometer. Films were measured on a Fluorolog-3 spectrofluorometer (FL 3-22, Jobin Yvon, Horiba) and excited by a Xenon lamp source. The emitted photons were collected in the front face geometry with a photomultiplier tube (PMT) R928P detector with a slit width of 5 nm.

Cyclovoltammetry experiments were performed with a Metrohm Autolab PGSTAT101 Electrochemical Analyser and spectra collected on NOVA software. The experiment was set up using a Ag/Ag^{+} reference electrode and a Pt wire counter electrode. The polymers were drop-casted on the glassy carbon working electrode and measurements taken at a scan rate of 0.1 V

s^{-1} with tetrabutylammonium hexafluorophosphate in acetonitrile (0.1 M) as the supporting electrolyte.

Molecular weight analyses were carried out on an **analytical GPC** Agilent Technologies 1200 series GPC equipped with a RI and UV detector running in chlorobenzene at 80°C, using two PL mixed-B columns in series. Narrow polydispersity standards were used to calibrate the system.

High-temperature GPC: High-temperature gel permeation chromatography (HT-GPC) measurements were carried out on an Agilent 1260 II High-Temperature GPC System fitted with three Agilent PLgel 10 µm MIXED-B, 7.5 x 300 mm columns, RID and HTELSD detectors, using HPLC-grade 1,2,4-trichlorobenzene (with 0.0125% BHT inhibitor) as eluent at a flow rate of 1.0 mL min^{-1} and temperature of 150°C. Number average molecular weight (M_n), weight average molecular weight (M_w), and dispersity (*Đ*) values are reported relative to poly(styrene) standards that were run at the same temperature. Samples were prepared to a concentration of approximately 1 mg mL^{-1} in the same solvent as the eluent.

Thermogravimetric analysis (TGA) was carried out using a Mettler Toledo TGA/DSC1, heating from 25 to 750°C at 5°C/min under N_2.

Differential scanning calorimetry was carried out using a Mettler Toledo DSC1.

GIWAXS diffractograms for Chapter 5 were obtained using a Xenocs Xeuss 3.0 SAXS/WAXS system equipped with an auxilliary Cu Kα Genix3D microfocus X-ray (1.54 Å) source and an Eiger2 4M detector. Films were prepared by spin-coating the polymer onto silicon substrates. Measurements were conducted under vacuum to suppress air scattering, and incidence angles were chosen which maximised scattering intensity. Data analysis and image processing was conducted in XSACT 2.0.

Photoelectron (UPS) and low energy inverse photoemission spectroscopy (LE-IPES) was performed in a ScientaOmicron X-ray Photoelectron (XPS)/Ultraviolet photoelectron spectroscopy (UPS) chamber with an adjoining LE-IPES chamber operating at 10^{-10} mbar. Samples were prepared by spin-coating on ITO and contacted to the sample plate with molybdenum strips for electrical contact with the analyzer. UPS was measured at an electron take-off angle normal to the sample surface with a 10 V bias applied between the ground to observe the secondary electron cutoff. A partially attenuated 21.2 eV vacuum UV (He 1 source) (HIS-13 focus) and an Argus CU electron analyzer were used. UPS was collected with a pass

energy of 3 eV. LE-IPES was measured after UPS. A homebuilt setup consisting of a 0.25 eV dispersion electron gun operating in the 20-30 eV range was used. Photons were first collected and focused with a lens in-vacuum assembly and then focused onto a Hamamatsu photomultiplier tube (PMT) with a bandpass filter of 280 nm (4.43 eV) (Semrock). A retarding bias of 20 eV was applied to the sample, and the current 0-10 μA was measured with a Keithley ammeter.

J-V measurements were conducted using a Keithley 4200 Source-Measure unit (scan rate 25 mV s^{-1}). An Oriel Instruments Solar Simulator with a Xenon lamp and calibrated to a silicon reference cell was used to provide AM1.5G irradiance. For determination of the Linear Dynamic Range (LDR), 780 nm LED driven by a function generator (ThorLabs DC2200) was used. The LED light was attenuated using a selection of neutral density filters placed between the LED and OPD. The photocurrent density (J_{ph}) was calculated as the difference in response between the illuminated current density (J_{light}) and dark current density (J_d) at each light intensity. All the devices were tested in air.

Responsivity was measured using an integrated system from Quantum Design PV300 with a modulation frequency of 90 Hz. All the devices were tested in ambient air.

Dynamic measurements were performed using a digital oscilloscope (Siglent, SDS6054A). The OPDs were illuminated with a 780 nm LED driven by a function generator (ThorLabs DC2200). For determination of the rise and fall time a 1kHz square wave pulse was applied to the LED using the function generator. For determination of the cut-off frequency sinusoidal functions with varying frequencies between 100 Hz and 1 MHz were used to drive the LED connected to a FEMTO-100 preamplifier. All the devices were tested in ambient air.

Charge carrier mobility measurement: The mobility of each pristine material and blends was measured using steady-state space-charge limited current (SCLC) method. In order to make sure device electrodes can inject the desired charge carrier while blocking the carrier with different polarity, hole-only (glass/ITO/PEDOT:PSS/active layer/Au) and electron-only (glass/ITO/ZnO/active layer/Ca/Al) devices were fabricated. Devices were placed in the sample chamber and then measured in the dark from 0V to 10 V under nitrogen environment.

2. Experimental

FO6 was prepared according to recently published protocol.[1]

Synthesis of 4,7-dibromo-5-fluoro-6-((2-hexyldecyl)oxy)benzo[c][1,2,5]oxadiazole (FO6-BO)

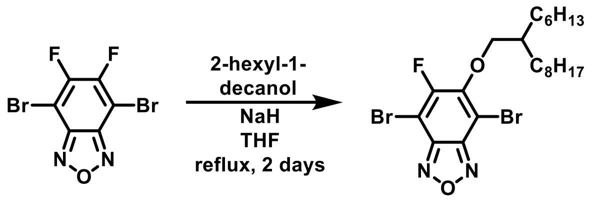

NaH (60% dispersion in mineral oil) was washed with hexane prior to use. The grey solid was dispersed in hexane and stirred for 10 minutes, after which the solid was filtered off and washed with another portion of hexane on the filter. The obtained powder was weighed for the reaction.

4,7-Dibromo-5,6-difluorobenzo[*c*][1,2,5]oxadiazole (1 g, 3.19 mmols, 1 eq.) and NaH (153 mg, 6.37 mmol, 2 eq.) were added to a 100 mL two-necked round bottom flask and flushed with nitrogen three times before dry THF (30 mL) was added via syringe. 2-Hexyl-1-decanol (0.77 g, 0.92 mL, 3.19 mmol, 1 eq.) was added via syringe and the mixture was heated to reflux while stirring for 2 days. Over the course of the reaction, the mixture turned brown. The reaction was allowed to cool to room temperature and transferred to a separating funnel with dichloromethane and brine and extracted with dichloromethane (3 x 50 mL). The combined organic layers (brown solution) were dried over Na_2SO_4, filtered and the solvent was removed under reduced pressure. The crude brown residue was purified using column chromatography with petroleum ether and ethyl acetate (30:1 v:v), followed by further purification by recycling GPC with CH_2Cl_2 to obtain the product as a light-yellow to colourless oil. Yield: 0.51 g (0.95 mmol, 30%). ^{1}H NMR (400 MHz, $CDCl_3$) δ 4.12 (d, J = 5.1 Hz, 2H), 1.88 – 1.77 (m, 1H), 1.62 – 1.50 (m, 2H), 1.50 – 1.38 (m, 22H), 0.89 (m, 6H). ^{19}F NMR (377 MHz, $CDCl_3$) δ -107.23. ^{13}C NMR (101 MHz, $CDCl_3$) δ 157.22 (d, J = 264 Hz), 150.95 (d, J = 20.76 Hz), 147.25, 146.30 (d, J = 6.04 Hz), 99.44 (d, J =3.02 Hz), 92.9 (d, J =27.21), 78.47 (d, J =5.03 Hz) , 39.20, 32.04, 31.98, 31.05, 30.09, 29.76, 29.72, 29.46, 26.93 (d, J = 2.27), 22.83 (d, J = 1.93), 14.26. HRMS (APCI): m/z calcd. for $C_{22}H_{34}FN_2O_2$[79]Br[81]Br^+ = 537.0945 $[M+H]^+$; found: 537.0949.

General procedure for the preparation of the polymers **FO6-T** and **FO6-BO-T**[1]

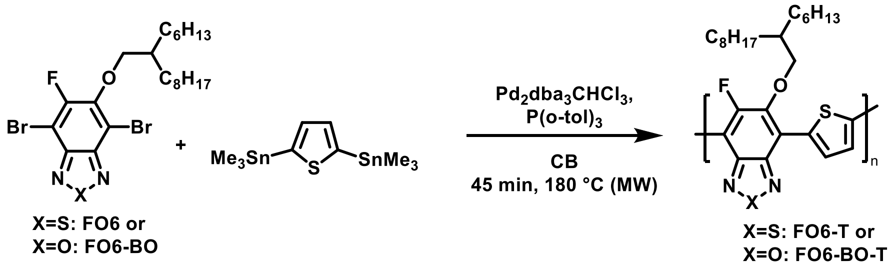

Both monomers (0.5 mmol, 1 eq.), $Pd_2(dba)_3{\cdot}CHCl_3$ (0.01 mmol, 0.02 eq.) and P(*o*-tol)$_3$ (0.04 mmol, 0.08 eq.) were placed in an oven-dried 20 mL microwave vial and flushed with nitrogen three times. Dry chlorobenzene (CB) (10 mL) was degassed for approx. 30 min and added to the reaction vial via syringe. The reaction mixture was degassed properly for 20 min. The vial was transferred to a microwave reactor and heated stepwise to 180°C (120°C for 2 min, 140°C for 2 min, 160°C for 2 min) and the temperature was kept for 40 min. Afterwards, the blue viscous solution was precipitated into methanol, followed by filtration to isolate the crude polymer. Purification was carried out using Soxhlet extraction with methanol, acetone, hexane and chloroform (in that order). The chloroform fraction was concentrated to approx. 10 mL and precipitated in methanol. The suspension was stirred for 20 min and the polymer was isolated as a blue solid.

FO6-T: 234 mg, 92%.

FO6-BO-T: Yield: 225 mg, 98%.

3. NMR Spectra

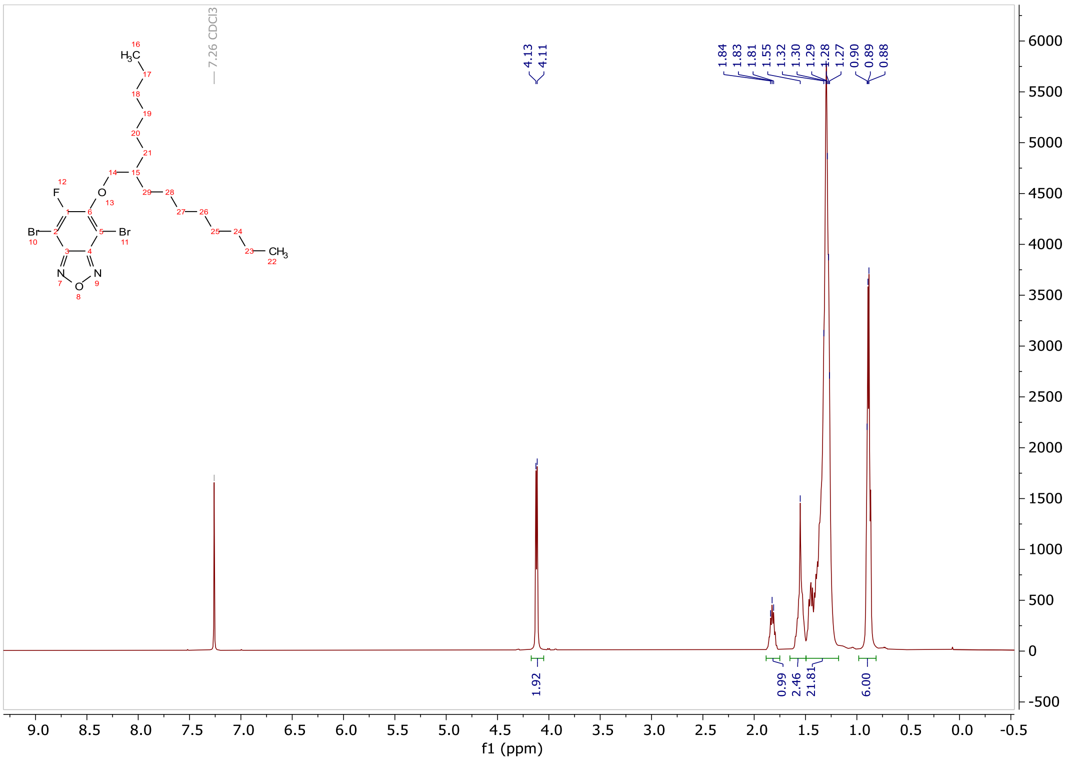

Figure S1. ^{1}H-NMR spectrum of **FO6-BO** in $CDCl_3$.

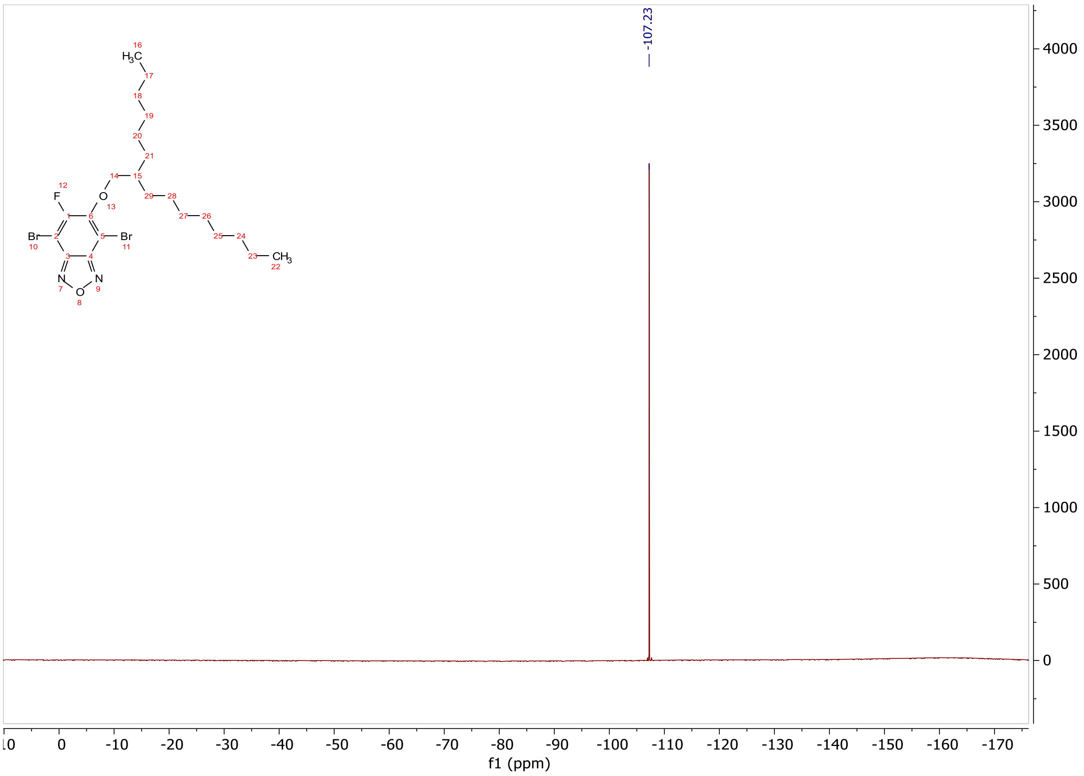

Figure S2. ^{19}F-NMR spectrum of **FO6-BO** in $CDCl_3$.

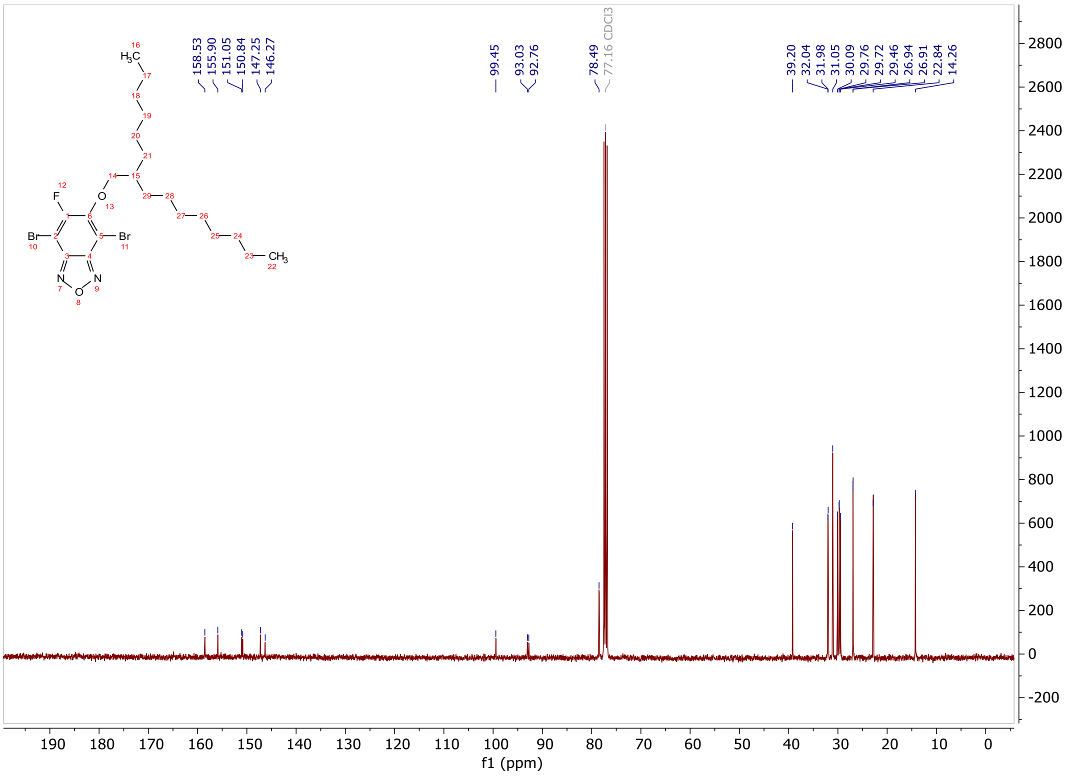

Figure S3. ^{13}C-NMR spectrum of **FO6-BO** in $CDCl_3$.

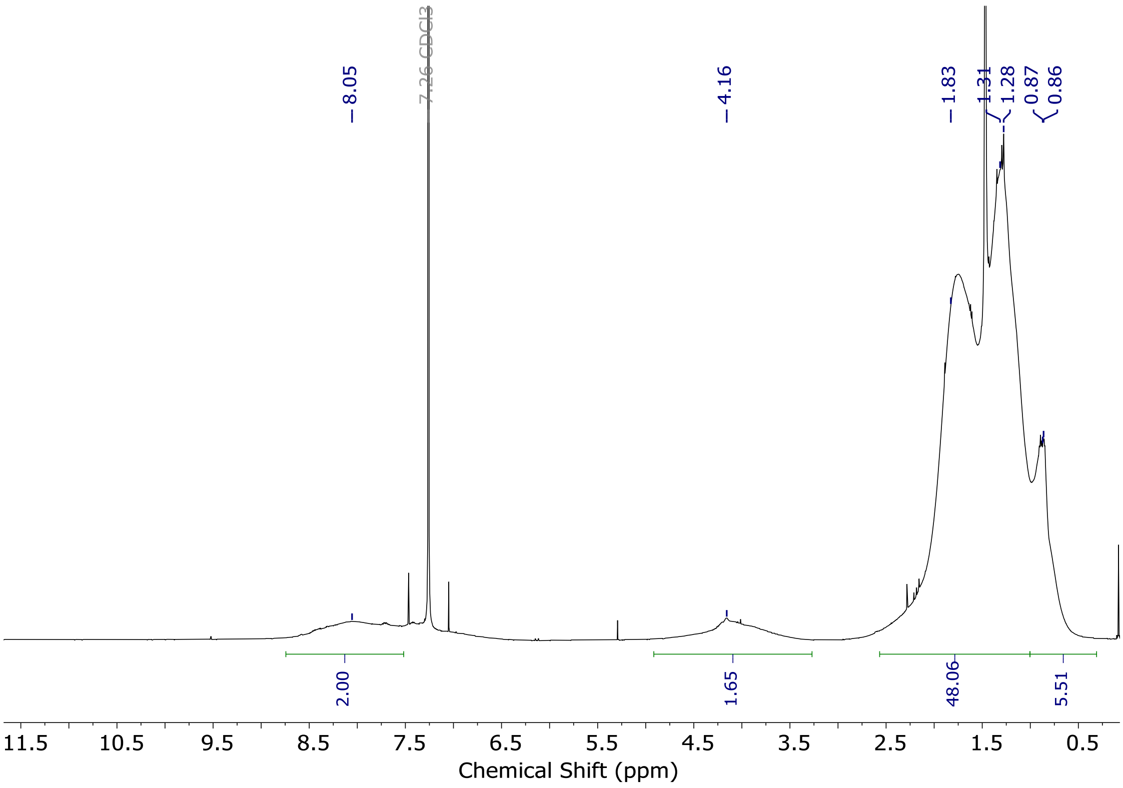

Figure S4. ^{1}H-NMR spectra of **FO6-BO-T** recorded in $CDCl_3$ at 55 °C.

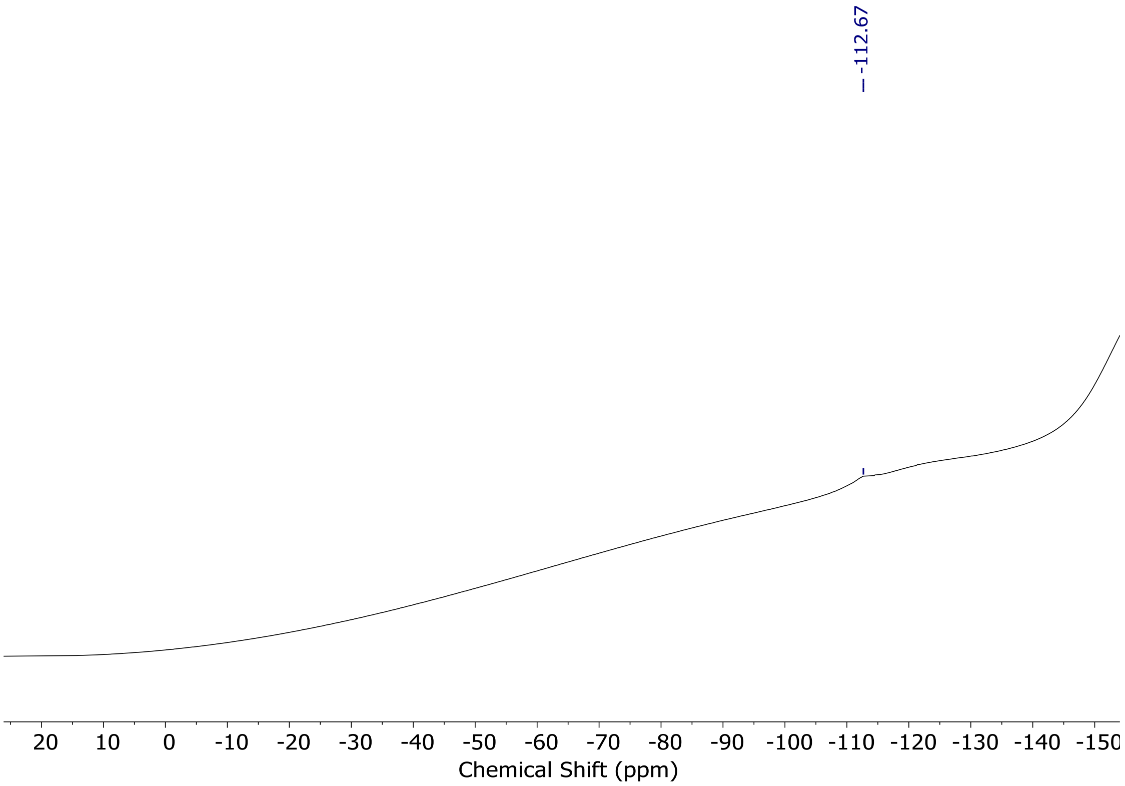

Figure S5. ^{19}F NMR spectra of **FO6-BO-T** recorded in $CDCl_3$ at 55 °C.

4. Molecular weight characterisation

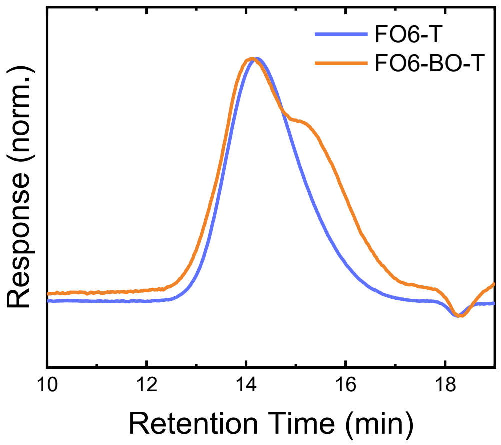

Figure S6. GPC traces of FO6-T and FO6-BO-T in 1,2,4-trichlorobenzene at 150°C.

5. Photoluminescence

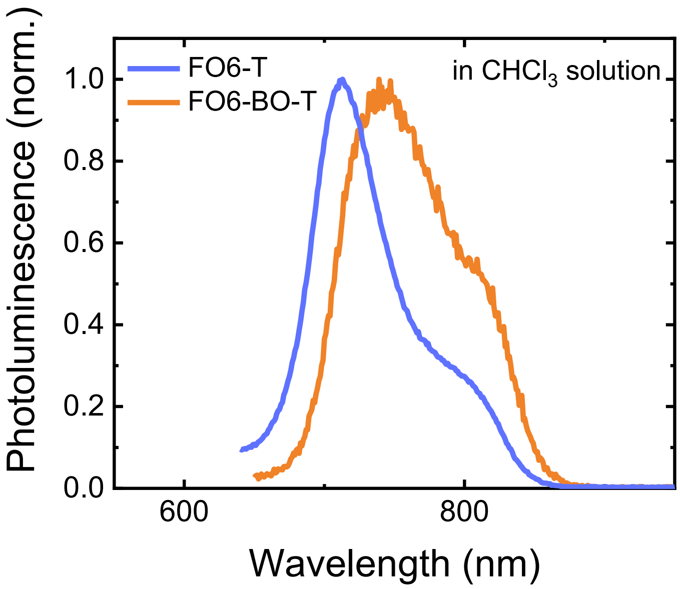

Figure S7. Normalised photoluminescence of FO6-T and FO6-BO-T in chloroform solution.

6. DFT

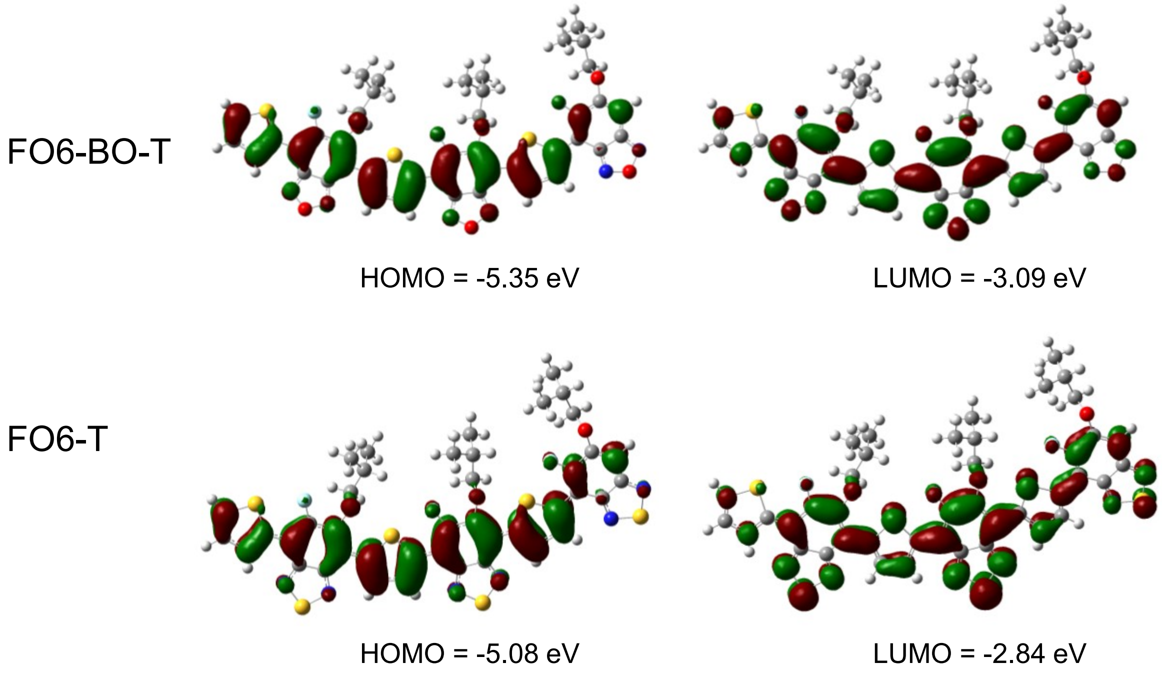

Figure S8. Frontier molecular orbitals at the HOMO and LUMO energy levels for the respective polymer.

7. UPS/LE-IPES

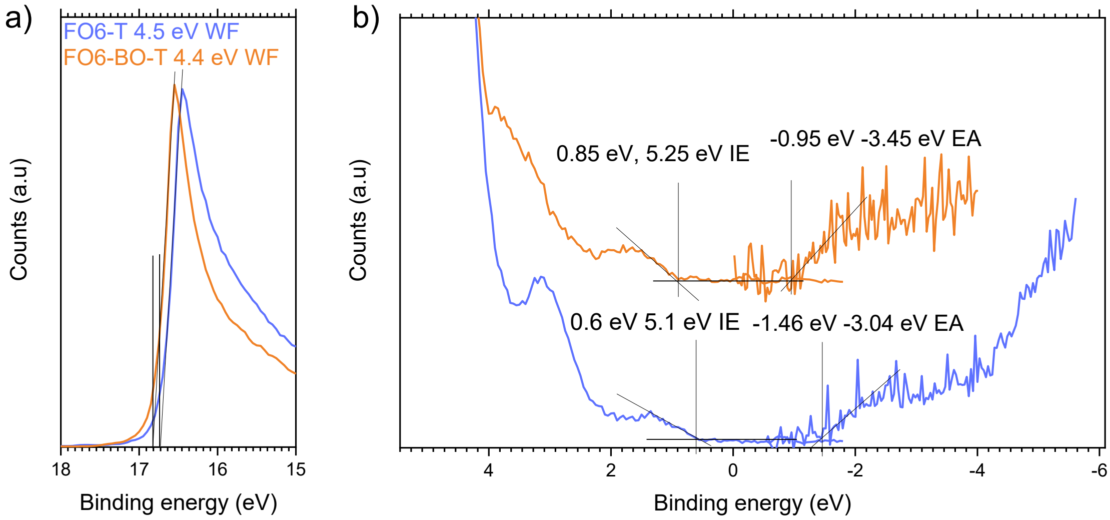

Figure S9. UPS measurement of FO6-BO-T and FO6-T thin films under vacuum. a) Vacuum level cut-off region for determination of Fermi energy. b) Valence band and photoelectron cut-off region utilizing UPS and IPES data. Ionisation energy is referenced to the vacuum level.

8. Thermal Characterisation

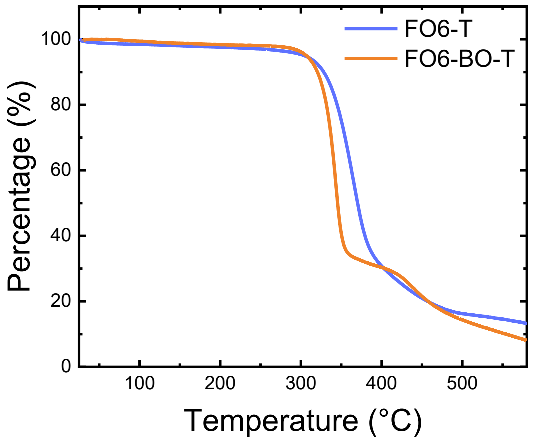

Figure S10. TGA Traces of FO6-T and FO6-BO-T.

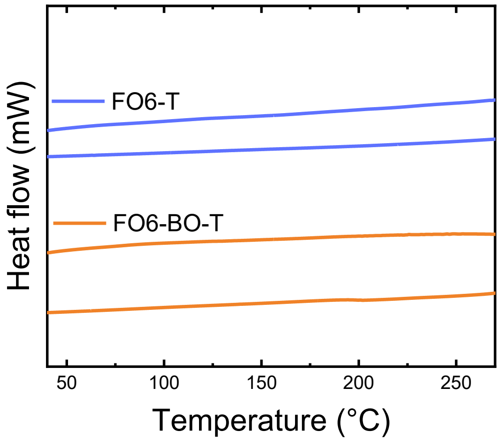

Figure S11. DSC traces of FO6-T and FO6-BO-T

Approximation of the glass transition temperature T_g via changes in the absorption spectra upon heating. The deviation metric is defined as following:

$$DM_T = \sum_{\lambda_{min}}^{\lambda_{max}} [I_{RT}(\lambda) - I_T(\lambda)]^2$$

$I_{RT}(\lambda)$ and $I_T(\lambda)$ are the normalised absorption intensities at room temperature and at annealing temperature T.

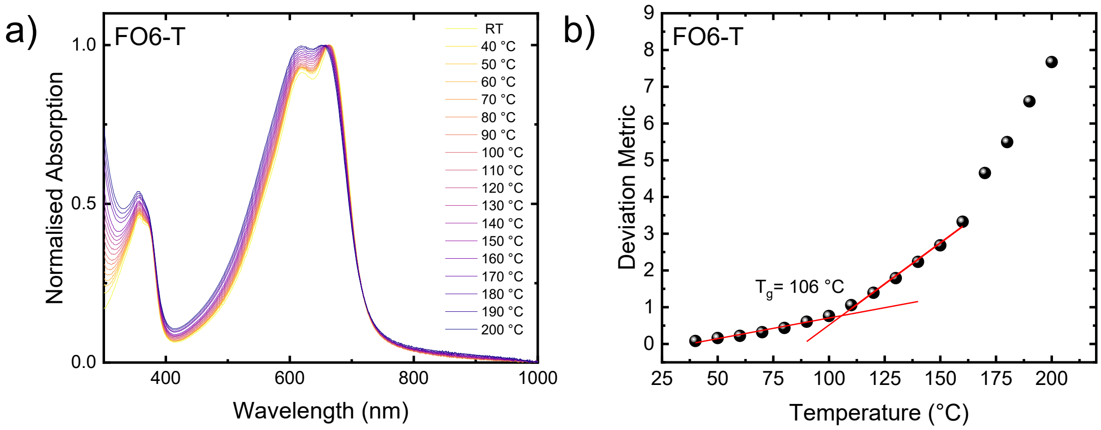

Figure S12. a) Normalised absorption spectra of FO6-T at RT and different annealing temperatures T. b) Deviation metric showing a distinct transition at T_g = 106°C.

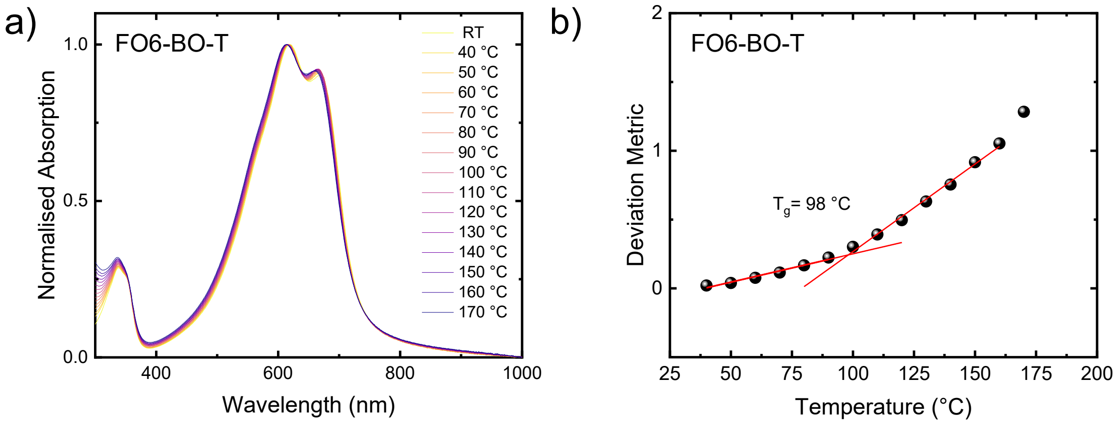

Figure S13. a) Normalised absorption spectra of FO6-BO-T at RT and different annealing temperatures T. b) Deviation metric showing a distinct transition at T_g = 98°C.

9. Device Fabrication

Organic photodetectors were produced in a cleanroom environment using an inverted structure (glass/ITO/ZnO/active layer/MoO_3/Ag). Indium tin oxide (ITO, 15 Ω sq^{-1}) was pre-patterned on 12 mm × 12 mm glass substrates. The substrates underwent a cleaning process involving sonication in acetone for 10 minutes, followed by detergent and deionised water. After an additional 10-minute sonication in acetone, the substrates were cleaned with isopropanol and immediately subjected to an 8-minute oxygen plasma treatment in a vacuum environment.

A 40 nm thick ZnO layer was deposited on the ITO through spin-coating at 4000 rpm for 40 seconds, using a zinc acetate dihydrate precursor solution. The coated substrates were then moved into a dry nitrogen glove box (with < 0.1 ppm H_2O and < 0.1 ppm O_2). The active layer solutions, comprising a blend of donor and acceptor IDSe in chloroform, were prepared in a 1:1.5 ratio (wt/wt) at a concentration of 25 mg/ml. The solutions were stirred overnight in the glove box at room temperature and heated to 40°C for 20 minutes before spin-coating to ensure complete dissolution.

The active layer solution was spin-coated onto the ZnO from the warm solution, under a nitrogen atmosphere, at spin speed of 1000 rpm for 40 seconds. The resulting active layers underwent thermal annealing (TA) treatment on a programmable heat plate in the glove box at 100°C for 10 minutes. For thermal evaporation, a 10 nm MoO_3 layer followed by a 100 nm Ag layer were sequentially deposited. The MoO_3 layer was deposited at a rate of 0.15 Å s^{-1}, and

the Ag layer deposition speed ranged from 0.5 Å s^{-1} to 1 Å s^{-1}. Each pixel's area, defined by a shadow mask, was 0.045 cm^2.

10. OPD Characterisation

D* is often reported in the literature using shot noise as i_n. Shot noise (i_{shot}), which is nominated by J_d, can be calculated from equation the following equation:

$$i_{shot} = \sqrt{2eJ_dA}$$

Where e is the elementary charge.

D* was determined using the calculated noise levels in order to provide a comparison to D* values for measured noise levels. We found higher (overestimated) values of $4.52{\cdot}10^{12}$ Jones for FO6-BO-T:IDSe and $3.36{\cdot}10^{12}$ Jones for FO6-T:IDSe.

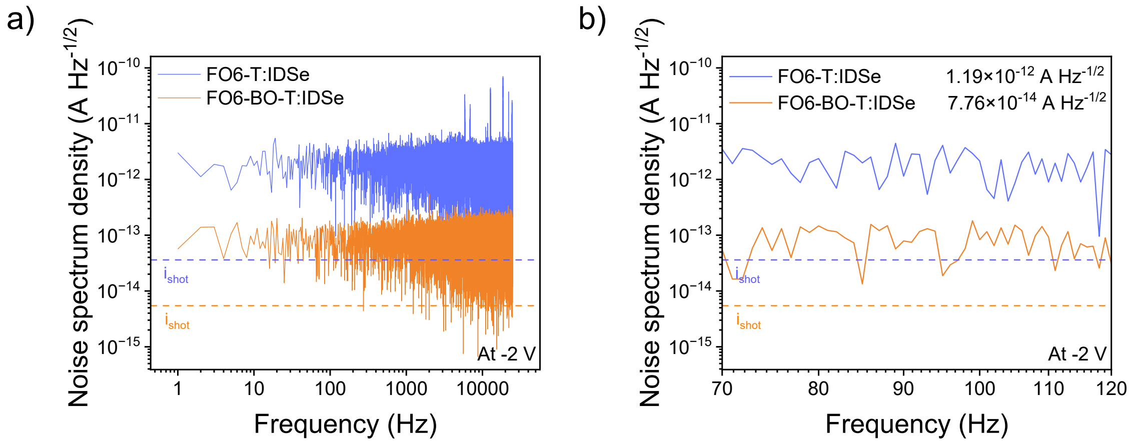

Figure S14. a) Noise spectral density for the respective polymer blends at -2 V, shot noise of each blend are indicated by dashed lines. b) Noise spectral density for the respective polymer blends at -2 V from 70 Hz to 120 Hz.

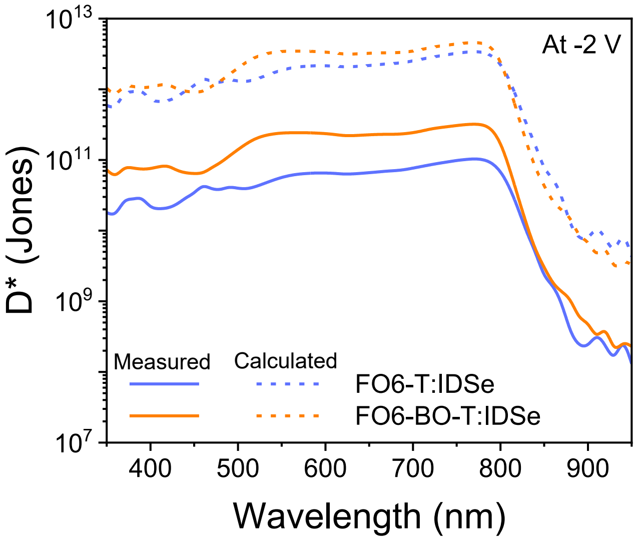

Figure S15. Comparison of specific detectivity of OPDs calculated by measured noise (solid line) and shot noise (dashed line) at -2 V.

11. Charge carrier mobilities

Table S1: Determined electron and hole mobilities for the polymers and their blends with IDSe.

	e^- mobility (cm^2/Vs)	h^+ mobility (cm^2/Vs)
**FO6-T**		$2.22{\cdot}10^{-4}$
**FO6-BO-T**		$6.21{\cdot}10^{-5}$
**FO6-T:IDSe**	$3.11{\cdot}10^{-5}$	$5.60{\cdot}10^{-5}$
**FO6-BO-T:IDSe**	$8.66{\cdot}10^{-6}$	$4.40{\cdot}10^{-6}$

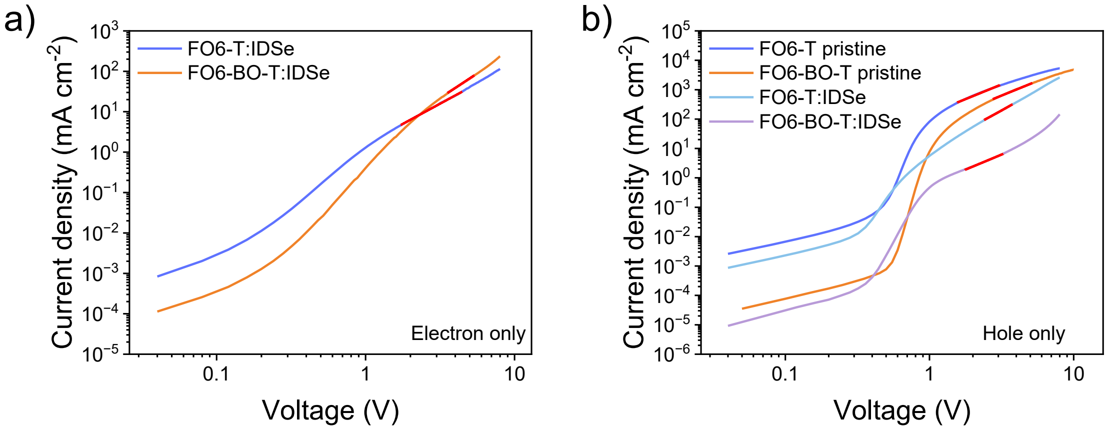

Figure S16. J-V curves and the linear fit for single charge carrier devices based on different polymers and their blends with the acceptor IDSe for the determination of a) electron mobilities and b) hole mobilities.

12. GIWAXS

Table S2: GIWAXS derived d-spacing calculated for neat and blend films of FO6-T, FO6-BO-T and IDSe

Sample peak		q (Å^{-1})	d (Å)
**FO6-T**	[100] (IP)	0.28	22.4
	[010] (OOP)	1.62	3.88
**FO6-T blend**	FO6-T [100]	0.28	22.4
	FO6-T [010]	1.61	3.90
	IDSe	0.42, 0.56	15.0, 11.2
**FO6-BO-T**	[100] (OOP)	0.30	20.9
	[010] (IP)	1.65	3.81
**FO6-BO-T blend**	FO6-BO-T [100]	0.28	22.4
	IDSe	0.41, 0.56	15.3, 11.2

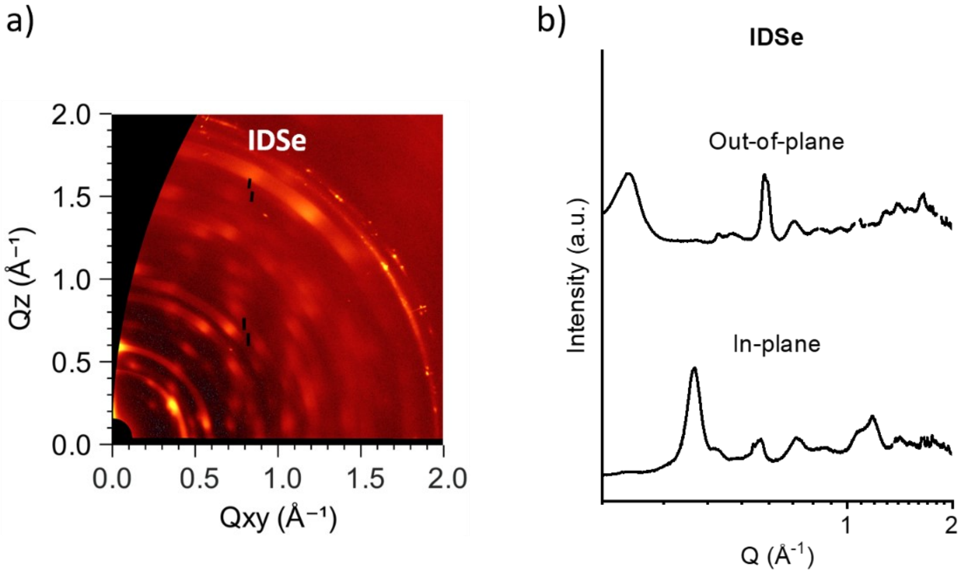

Figure S17. a) 2D GIWAXS image and b) 1D line plot of a pristine IDSe film.

13. References

1. Rimmele, M.; Qiao, Z.; Panidi, J.; Furlan, F.; Lee, C.; Tan, W. L.; McNeill, C. R.; Kim, Y.; Gasparini, N.; Heeney, M., A polymer library enables the rapid identification of a highly scalable and efficient donor material for organic solar cells. *Materials Horizons* **2023,** *10* (10), 4202-4212.
